# Microbiological profile of prosthetic joint infections in orthopedic oncology: a comparison with conventional joint arthroplasty

**DOI:** 10.5194/jbji-10-459-2025

**Published:** 2025-11-18

**Authors:** Joseph J. Connolly, Marcos R. Gonzalez, Joshua B. Davis, Youssef H. Moussaoui, Graham S. Goh, Antonia F. Chen, Adam S. Olsen, Santiago A. Lozano-Calderón

**Affiliations:** 1 Orthopaedic Oncology Service, Department of Orthopaedic Surgery, Massachusetts General Hospital, Harvard Medical School, Boston, MA 02114, USA; 2 Division of Arthroplasty and Joint Reconstruction, Department of Orthopaedic Surgery, Brigham and Women's Hospital, Harvard Medical School, Boston, MA 02115, USA; 3 Department of Orthopaedic Surgery, Boston University Medical Center, Boston, MA 02118, USA; 4 Department of Orthopaedic Surgery, University of Texas Southwestern Medical Center, Dallas, TX 75390, USA

## Abstract

**Background**: Periprosthetic joint infections (PJIs) are a devastating complication following oncologic endoprosthetic reconstruction (EPR). Despite significant efforts to characterize the microbiologic profile of PJI in traditional joint arthroplasty, data are lacking in orthopedic oncology. Our study analyzed the causative microorganisms and time to positivity (TTP) of PJI in oncologic EPR and conventional joint arthroplasty (C-TJA). **Methods**: We retrospectively compared sample cultures for lower-extremity oncologic EPR and C-TJA patients diagnosed with PJI between 2000 and 2022. All positive microorganisms were assessed, along with clinical and culture method data. Comparisons utilized the Mann–Whitney U test. **Results**: We included 70 oncologic EPR and 153 C-TJA patients diagnosed with PJIs. *Staphylococcus epidermidis* (16.8 % vs. 10.6 %, p=0.01 ), *Enterococcus* spp. (12.6 % vs. 4 %, p<0.001 ), and *Peptostreptococcus* spp. (5.3 % vs. 1.3 %, p<0.001 ) were common and more frequently isolated in oncologic EPR than C-TJA PJI. Conversely, *Staphylococcus aureus* predominated in samples from C-TJA patients (31.7 % vs. 15.1 %, p<0.001 ). Differences in endoprosthetic microorganism prevalence were observed between primary versus metastatic bone disease and bone versus soft tissue sarcoma. TTP was highly variable among microorganisms and was significantly faster ( p<0.05 ) for bone and soft tissue vs. synovial fluid (3 d vs. 4 d) and for broth and solid media vs. broth only (2.5 d vs. 4.5 d). **Conclusion**: The microorganism profile in oncologic EPR PJI was distinct from C-TJA PJI. The oncologic EPR population highlighted variability in the prevalence of Gram-negative rods and slower TTP for broth-only cultures. Further investigation of the mechanisms behind these differences will allow care teams to provide prompt, individualized, and targeted antimicrobial therapy.

## 1 Introduction

The use of endoprosthetic reconstruction (EPR) in patients with bone tumors is a well-established treatment option that allows limb salvage and restoration of function in the affected joint. Despite its success, periprosthetic joint infection (PJI) remains a devastating complication with serious clinical and economic implications (Kurtz et al., 2012; Premkumar et al., 2021). In orthopedic oncology, the prevalence of PJIs is higher than in primary total joint arthroplasty for non-oncologic indications (7 %–28 % vs. 1 %–2 %, respectively), and PJI is the most common mode of failure following oncologic EPR (Henderson et al., 2011; Zuidhof et al., 2019). These figures are not surprising considering the myriad of risk factors in oncologic patients, including chronic immunodeficiency, adjuvant radiation therapy (RT) and chemotherapy, and greater surgical complexity (e.g., wider exposure, increased surgical duration) (Zuidhof et al., 2019). Despite these additional risk factors, current International Consensus Meeting (ICM) 2018 recommendations for the management of PJI in orthopedic oncology do not differ from those in other orthopedic subspecialties.

In recent years, the concept of the host microbiome and its role in the development of periprosthetic joint infection has gained popularity (Abdeen et al., 2022; Carr et al., 2021; Fernández-Rodríguez et al., 2023; Hernandez, 2021; Torchia et al., 2020; Torrens et al., 2022; Zuidhof et al., 2019). Parvizi and colleagues recently found that the human knee microbiome is distinct and may predispose certain patients to PJI (Fernández-Rodríguez et al., 2023). Preclinical models have also demonstrated that an altered microbiome composition can increase the risk of PJI in mice (Hernandez et al., 2019). Moreover, some authors have coined the “Trojan Horse” theory, wherein gut dysbiosis and increased intestinal permeability have been suggested to increase the risk of PJI (Chisari et al., 2022a, b; Hernandez, 2021). In tandem with these investigations, several oncologic studies have linked gut dysbiosis to cancer development, suggesting that oncologic patients may have a distinct microbiome (Le et al., 2023; Wang et al., 2017). Furthermore, several studies – primarily in gastrointestinal malignancies but also in sarcoma – have identified microbiome-driven carcinogenesis pathways, while others have gone on to identify biomarkers and potential treatment strategies for anti-cancer therapy (Boursi et al., 2015; Li et al., 2012; Perry et al., 2023; Stoff et al., 2023; Veziant et al., 2021; Wang et al., 2017; Wong and Yu, 2019; Yu et al., 2017). It follows that if the host microbiome is altered in oncologic patients, the biological environment of the resected joint and subsequent infecting microorganism for PJI after oncologic EPR may differ. While recent studies have sought to evaluate the microbiologic profile of PJI in traditional, non-oncological joint arthroplasty, limited data are available for oncologic patients with oncologic EPR (McCulloch et al., 2022; Tarabichi et al., 2023; Weinstein et al., 2023).

The purpose of this study was to characterize the microbiologic profile of oncologic patients who developed a PJI after undergoing lower-extremity oncologic EPR and compare it to that of patients undergoing conventional arthroplasty for end-stage arthritis. A deeper understanding of the microbial composition and relative frequencies of causative organisms in oncologic endoprosthetic PJI could allow more timely, targeted antimicrobial treatment of PJI in these immunocompromised patients who are particularly at risk of uncontrolled sepsis and of systemic toxicity related to broad spectrum antimicrobials.

## 2 Methods

### 2.1 Patient inclusion

Institutional review board approval was obtained prior to the start of this study. We conducted a retrospective review of a prospectively maintained institutional endoprosthesis registry. We included patients who underwent primary tumor resection and EPR around the hip or knee joint and subsequently developed a PJI between 2000 and 2022 at a large tertiary academic center. PJI was defined using the 2011 Musculoskeletal Infection Society (MSIS) criteria (Parvizi et al., 2011). Patients with endoprostheses not involving the hip or knee joint, or with prior aseptic revisions before the PJI, were excluded. Out of 90 patients with endoprosthetic PJIs, we excluded 20 whose endoprostheses were implanted for non-oncologic indications and 5 whose infections did not involve the hip or knee, resulting in a final oncologic EPR cohort of 70 patients.

For the conventional arthroplasty group, a PJI arthroplasty registry from a large tertiary care academic center was queried. We only included patients who had a PJI after a conventional primary total hip arthroplasty (THA) or total knee arthroplasty (TKA) for arthritic indications. Patients with PJI following revision arthroplasty for non-infectious indications were excluded, along with those without culture data. Out of 230 eligible patients with MSIS-confirmed PJI, we excluded 77 patients due to prior revisions or unavailable culture data. This resulted in a final sample of 153 patients with conventional implant PJIs.

### 2.2 Study variables

A manual chart review of each patient's medical record was performed to confirm the number of samples collected, specimen type (i.e., bone, soft tissue, synovial fluid), isolated pathogen at final reporting, and time to positivity (TTP) for each pathogen in each sample cultured. Surgical and oncologic data were collected, including the operated joint, laterality, tumor type (i.e., primary bone tumor vs. metastatic bone lesion), and endoprosthesis type. Patient demographics were also recorded, including age, sex, body mass index (BMI), age-adjusted Charlson Comorbidity Index, American Society of Anesthesiologists (ASA) score, and host grade determined according to the McPherson score (McPherson et al., 2002).

For each case, between three and eight intraoperative samples were collected and sent for aerobic, anaerobic, fungal, and mycobacterial cultures. Per institutional practice, perioperative antibiotic prophylaxis was administered only after cultures were obtained. This comprised cefazolin 1–2 g and vancomycin 15 mg kg^−1^ or clindamycin 900 mg. Acute PJI was defined as PJI occurring within 4 weeks of the procedure, and PJI presenting any time thereafter constituted a chronic PJI (Pellegrini et al., 2022). At our institution, samples are routinely cultured on solid media – consisting of a combination of blood, chocolate, and MacConkey agar plates – and thioglycolate broth. Samples are incubated at 37 °C in a Thermo Scientific Large-Capacity Reach-In CO_2_ Incubator (Model #3950). Aerobic and anaerobic cultures are routinely held for 14 d, while fungal and mycobacterial cultures are held for 28 and 56 d, respectively.

### 2.3 Study outcomes

The primary outcome was the frequency and proportion of each microorganism isolated in the oncologic EPR and conventional arthroplasty cohorts, respectively. Dual secondary outcomes included (1) the proportions of each microorganism in the different oncologic subpopulations (bone sarcoma, soft tissue sarcoma, and metastatic bone disease) and (2) the TTP for the oncologic EPR microorganisms based on Gram staining, culture origin, and incubation medium. For both groups, only cultures from the first septic revision were analyzed.

For all comparisons of proportions, the denominator was the total count of positive microorganism isolates within each group. In the case of polymicrobial culture samples, each microorganism was counted as a separate event.

### 2.4 Patient characteristics

A total of 70 patients were included in the oncologic EPR cohort, and 153 were included in the conventional arthroplasty cohort. The median age was 56 (IQR: 47, 68) and 67 (59, 73) years ( p<0.001 ), and 57.1 % and 34.6 % of patients were women ( p=0.002 ), respectively (Table 1).

**Table 1:** Demographics and clinical characteristics of the patients included in our study. Bolded values indicate statistically significant differences ( p<0.05 ).

	Endoprosthesis	Conventional	p value
	( N=70 )	( N=153 )	
Age^1^	56 (47, 68)	67 (59, 73)	< **0.001**
Female sex	40 (57.1 %)	53 (34.6 %)	**0.002**
BMI ≥ 30	27 (40.3 %)	74 (50.3 %)	0.17
ASA class^2^			**0.001**
2	37 (55.2 %)	46 (30.3 %)	
3	30 (44.8 %)	101 (66.4 %)	
4	0 (0.0 %)	5 (3.3 %)	
Age-adjusted CCI^1^	5 (3, 8)	4 (3, 6)	**0.009**
Host grade (McPherson score)			< **0.001**
A	9 (12.9 %)	55 (35.9 %)	
B	59 (84.3 %)	82 (53.6 %)	
C	2 (2.9 %)	16 (10.5 %)	
Tumor type			
Primary bone tumor	59 (84 %)		
Metastatic bone lesion	11 (16 %)		
Previous RT to area	34 (48.6 %)	0 (0.0 %)	< **0.001**

ASA: American Society of Anesthesiologists; BMI: body mass index; CCI: Charlson Comorbidity Index; RT: radiation therapy. ^1^ Values refer to the median and interquartile range (IQR) in parentheses. ^2^ Data on ASA class were not available for three patients in the oncologic endoprosthesis cohort and one patient in the conventional arthroplasty cohort.

All oncologic EPR patients had an ASA score of either II (55 %) or III (45 %), while conventional arthroplasty patients had scores of II (30.3 %), III (66.4 %), and IV (3.3 %) ( p=0.001 ). The cohorts differed based on the McPherson scoring system ( p<0.001 ); 12.9 % of oncologic EPR patients were host grade A, 84.3 % of these patients were host grade B, and 2.9 % of these patients were host grade C, while 35.9 % of conventional arthroplasty patients were host grade A, 53.6 % of these patients were host grade B, and 10.5 % of these patients were host grade C. From an oncologic perspective, 84 % of surgeries were indicated for a primary bone or soft tissue tumor, while 16 % of surgeries were indicated for metastatic bone disease. Almost half (48.6 %) of patients had previous RT to the operated area. PJI was classified as acute in 42.9 % and 44.4 % of oncologic EPR and conventional arthroplasty patients, respectively (Table 2). The most common types of EPR were distal femoral replacement (44 %) and proximal femoral replacement (33 %). TKA (78.4 %) was more common than THA (21.6 %).

**Table 2:** Infection and prosthesis characteristics of patients included.

	Endoprosthesis	Conventional	p value
	( N=70 )	( N=153 )	
PJI classification			0.82
Acute	30 (42.9 %)	68 (44.4 %)	
Chronic	40 (57.1 %)	85 (55.6 %)	
CRP (mg dL^−1^)^*^	108 (36, 146)	88 (29, 181)	0.76
ESR (mm h^−1^)^*^	58 (37, 98)	57 (32, 82)	0.21
Type of prosthesis			
PFR	23 (33 %)	–	
DFR	31 (44 %)	–	
TFR	7 (10 %)	–	
PTR	5 (7 %)	–	
DFR + PTR	4 (6 %)	–	
THA	–	33 (21.6 %)	
TKA	–	120 (78.4 %)	

CRP: C-reactive protein; DFR: distal femur replacement; ESR: erythrocyte sedimentation rate; PFR: proximal femur replacement; PTR: proximal tibia replacement; TFR: total femur replacement; THA: total hip arthroplasty; TKA: total knee arthroplasty. ^*^ Values refer to the median and interquartile range in parentheses.

### 2.5 Data analyses

Demographic and clinical characteristics were displayed using descriptive statistics. Normal distribution of data was assessed using the Shapiro–Wilk test. The median and interquartile range (IQR) were used for continuous variables due to non-normal distribution. Differences in continuous variables were compared using the Mann–Whitney U test (non-parametric), while those in categorical variables were compared using the $\chi^{2}$ test. A p value of <0.05 was considered statistically significant. All statistical analyses were performed using Stata (StataCorp) and the Anaconda Distribution (Continuum Analytics) with Python Version 3.6 (Python Software Foundation).

## 3 Results

**Figure 1:**
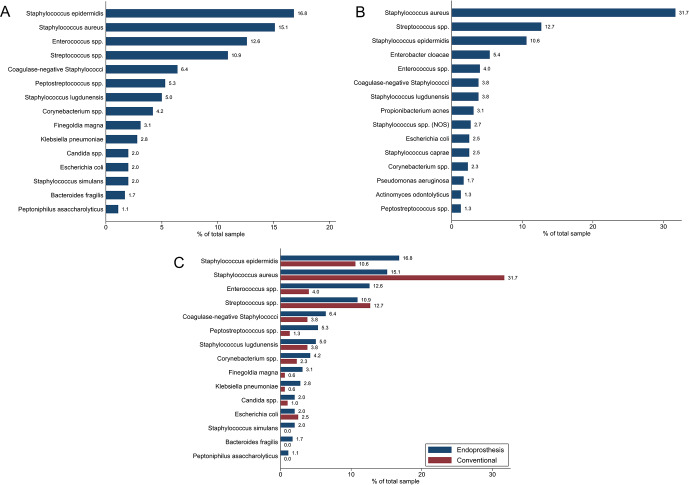
Proportion of each isolated microorganism in **(a)** the oncologic endoprosthetic reconstruction (EPR) cohort and in **(b)** the conventional arthroplasty cohort. **(c)** Comparison of microorganism proportions of oncologic EPR to those of conventional arthroplasty.

A total of 358 and 479 microorganism isolates were identified in the oncologic EPR and conventional arthroplasty cohorts, respectively. The three most prevalent pathogens isolated in cultures from oncologic EPR were *Staphylococcus epidermidis* ( n=60 , 16.8 %), *Staphylococcus aureus* ( n=54 , 15.1 %), and *Enterococcus* spp. ( n=45 , 12.6 %) (Fig. 1a). For conventional arthroplasty patients, the most commonly identified pathogens were *Staphylococcus aureus* ( n=165 , 31.7 %), *Streptococcus* spp. ( n=61 , 12.7 %), and *Staphylococcus epidermidis* ( n=51 , 10.6 %) (Fig. 1b).

*Staphylococcus epidermidis* ( p=0.01 ), *Enterococcus* spp. ( p<0.001 ), *Peptostreptococcus* spp. ( p<0.001 ), *Finegoldia magna* ( p=0.006 ), *Klebsiella pneumoniae* ( p=0.01 ), *Staphylococcus simulans* ( p=0.002 ), *Bacteroides fragilis* ( p=0.004 ), and *Peptoniphilus asaccharolyticus* ( p=0.02 ) were more frequently isolated in oncologic EPR samples compared to conventional arthroplasty ones (Fig. 1c). Conversely, *Staphylococcus aureus* was more frequently isolated in samples from conventional arthroplasty patients ( p<0.001 ).

**Figure 2:**
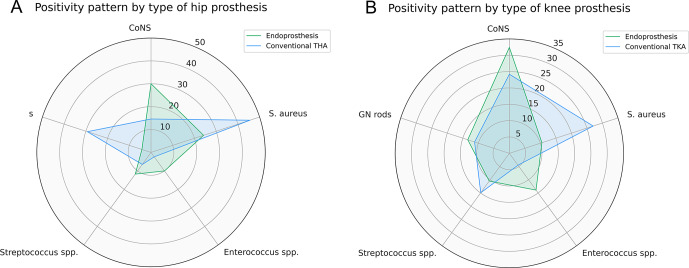
Comparison of the five most common microbiology groups in the endoprosthetic reconstruction and conventional arthroplasty cohort for prostheses around the **(a)** hip joint and **(b)** knee joint.

After stratifying by joint, hip endoprostheses had a higher frequency of coagulase-negative *Staphylococcus* (CoNS) (30 % vs. 14.6 %, p=0.004 ) and *Enterococcus* spp. (10 % vs. 2.4 %, p=0.01 ) and a lower frequency of *Staphylococcus aureus* (24.2 % vs. 45.5 %, p<0.001 ) and Gram-negative rod isolates (4.2 % vs. 29.3 %, p<0.001 ) compared to conventional THAs (Fig. 2a). Similarly, knee endoprostheses had a higher frequency of CoNS (32.4 % vs. 24.2 %, p=0.02 ) and *Enterococcus* spp. (13.9 % vs. 4.5 %, p<0.001 ) and a lower frequency of *Staphylococcus aureus* (10.5 % vs. 27 %, p<0.001 ) compared to conventional TKAs (Fig. 2b).

**Figure 3:**
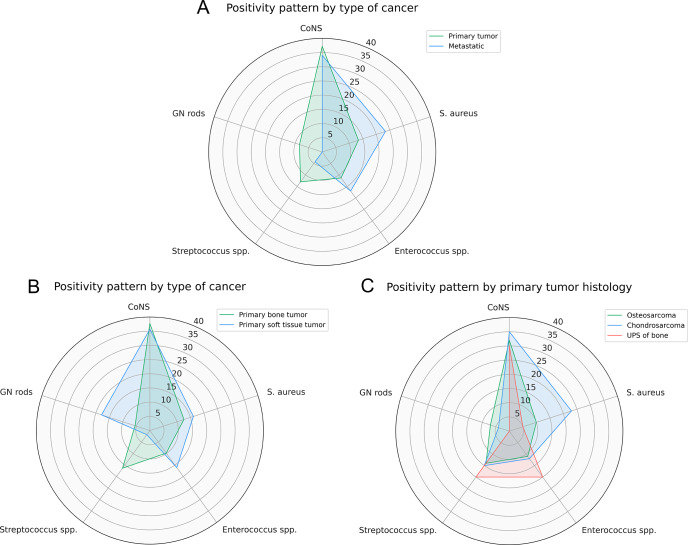
Prevalence of the five most common microorganisms stratified by **(a)** type of cancer (primary bone/soft tissue tumor vs. metastatic bone disease), **(b)** type of primary tumor (bone vs. soft tissue sarcoma), and **(c)** primary tumor histology (osteosarcoma vs. chondrosarcoma vs. UPS of bone).

A higher frequency of Gram-negative rod isolates was found in patients with EPR for primary sarcomas compared to those with metastatic bone disease (8.4 % vs. 0 %, p=0.04 ) (Fig. 3a). Among patients with primary sarcomas, patients with bone sarcomas demonstrated a higher frequency of *Streptococcus* spp. (16.4 % vs. 1.8 %, p=0.004 ) and a lower frequency of Gram-negative rod (5.5 % vs. 17.9 %, p=0.003 ) isolated compared to patients with soft tissue sarcomas (Fig. 3b). Within the primary bone sarcoma group, we did not observe any differences in the distribution of microorganisms by histology (Fig. 3c).

**Figure 4:**
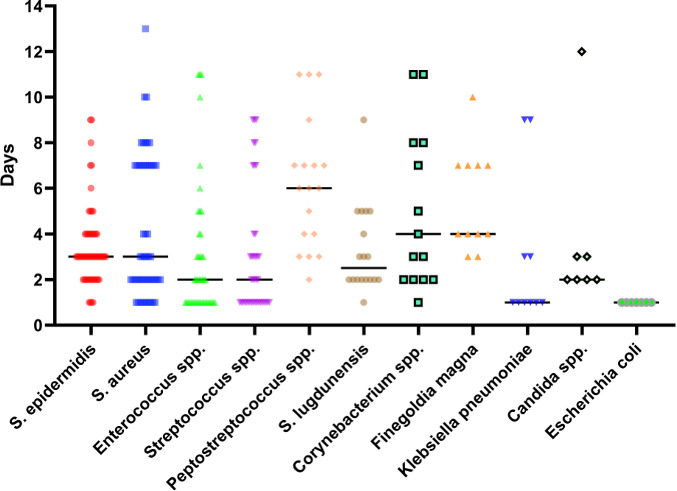
Time to positivity, in days, for selected common infective microorganisms in the oncologic endoprosthetic reconstruction cohort.

In the oncologic EPR cohort, *Escherichia coli* (1 d, IQR 1–1) and *Klebsiella pneumoniae* (1 d, IQR 1–3) had the fastest median TTP in days, followed by *Enterococcus* spp. (2 d, IQR 1–4), *Streptococcus* spp. (2 d, IQR 1–4), and *Candida* spp. (2 d, IQR 2–3) (Fig. 4).

The TTPs for rarer pathogens are shown in Table 3, which revealed notably slower TTPs for CoNS (8 d, IQR 5–15), *Peptostreptococcus* spp. (6 d, IQR 4–9), and *Bacteroides fragilis* (11 d, IQR 3–11).

**Table 3:** Time to positivity (TTP) of isolated microorganisms in the endoprosthesis cohort.

Microorganism	TTP (days)^*^
*Staphylococcus epidermidis*	3 (2, 4)
*Staphylococcus aureus*	3 (2, 7)
*Enterococcus* spp.	2 (1, 4)
*Streptococcus* spp.	2 (1, 4)
Coagulase-negative *Staphylococci*	8 (5, 15)
*Peptostreptococcus* spp.	6 (4, 9)
*Staphylococcus lugdunensis*	2.5 (2, 5)
*Corynebacterium* spp.	4 (2, 8)
*Finegoldia magna*	4 (4, 7)
*Klebsiella pneumoniae*	1 (1, 3)
*Candida* spp.	2 (2, 3)
*Escherichia coli*	1 (1, 1)
*Staphylococcus simulans*	2 (1, 3)
*Bacteroides fragilis*	11 (3, 11)
*Peptoniphilus asaccharolyticus*	5 (3, 5)

^*^ Values refer to the median and interquartile range in parentheses.

**Figure 5:**
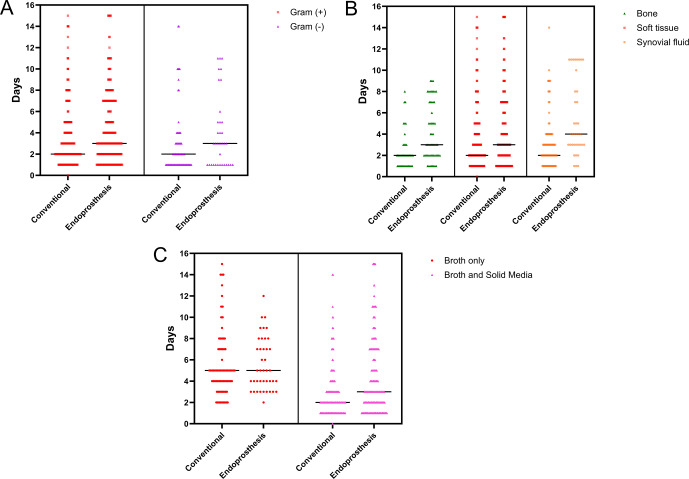
Comparison of time to positivity, in days, between the oncologic endoprosthetic reconstruction cohort and the conventional arthroplasty cohort **(a)** for Gram-positive vs. Gram-negative bacteria, **(b)** stratified by sample origin (bone, soft tissue, and synovial fluid), and **(c)** stratified by broth only vs. broth and solid media.

Within the oncologic EPR cohort, Gram-positive and Gram-negative bacteria had an equivalent median TTP of 3 d ( p=0.83 ) (Fig. 5a). When stratifying TTP based on culture origin, synovial fluid ( n=37 ; 4 d, IQR 3–8) demonstrated a significantly slower median TTP ( p=0.03 ) than bone ( n=58 ; 3 d, IQR 2–7) and soft tissue ( n=263 ; 3 d, IQR 2–7) (Fig. 5b). Samples cultured in solid media and broth had a significantly faster TTP ( n=315 ; 3 d, IQR 2–7; p<0.001 ) than samples cultured in broth only ( n=43 ; 5 d, IQR 3–8) (Fig. 5c).

**Table 4:** Numbers of samples included in the time-to-positivity comparison for Gram positivity, sample origin, and culture medium.

	Endoprosthesis	Conventional
	( N=358 )	( N=479 )
Gram positivity^*^	( N=349 )	( N=474 )
Gram-positive	312 (89.4 %)	398 (84.0 %)
Gram-negative	37 (10.6 %)	76 (16.0 %)
Sample origin		
Soft tissue	263 (73.5 %)	352 (73.5 %)
Bone	58 (16.2 %)	42 (8.8 %)
Synovial fluid	37 (10.3 %)	85 (17.7 %)
Culture medium		
Broth and solid media	315 (88.0 %)	405 (84.6 %)
Broth only	43 (12.0 %)	74 (15.4 %)

^*^ Data on Gram positivity were not available or applicable for nine patients in the oncologic endoprosthesis cohort and five patients in the conventional arthroplasty cohort.

When comparing TTP between oncologic EPR and conventional arthroplasty (Table 4), cultures from endoprostheses showed significantly slower TTP ( p<0.05 ) than conventional implants after stratifying by Gram positivity (Fig. 5a), culture origin (Fig. 5b), and type of media (Fig. 5c). The only exception was for microorganisms that grew exclusively in broth, where no difference was observed between types of prostheses.

## 4 Discussion

PJI remains an important cause of failure following EPR in oncologic patients, as these patients are at a higher risk of infection compared to conventional arthroplasty patients. Over the past decade, the concept of the human microbiome and its role in the pathogenesis of various diseases has permeated the medical literature. Importantly, multiple studies have linked an altered microbiome makeup to the development of both PJI and oncogenesis, highlighting the need to characterize the microbiologic profile of PJI following EPR for oncologic indications (Abdeen et al., 2022; Boursi et al., 2015; Carr et al., 2021; Contino et al., 2022; Fernández-Rodríguez et al., 2023; Li et al., 2012; Linke et al., 2022; Perry et al., 2023; Wang et al., 2017; Yuan et al., 2022). To our knowledge, this is the first study to suggest differences in the microbiologic profile and TTP of microorganisms causing PJI in oncologic EPR and conventional arthroplasty populations.

The most abundant microorganism species identified from oncologic EPR infections were *Staphylococcus epidermidis*, *Staphylococcus aureus*, *Enterococcus* spp., and *Streptococcus* spp., which overlaps considerably with the conventional arthroplasty cohort and falls in line with previously reported prevalence rates in conventional arthroplasty populations (Benito et al., 2019; Linke et al., 2022; Tande and Patel, 2014; Tarabichi et al., 2023). However, in contrast with a previous review of non-oncologic PJI studies, wherein CoNS and *Staphylococcus aureus* were most prevalent, our study found a much higher proportion of CoNS (including *Staphylococcus epidermidis*) compared to *S. aureus*. Additionally, *Enterococcus* spp. was seen in our oncologic EPR population in greater proportion than in our conventional arthroplasty population and previous non-oncologic PJI studies (Benito et al., 2019; Tande and Patel, 2014). In fact, *Enterococcus* spp. was not reported in some non-oncologic studies due to its apparent limited role (Linke et al., 2022; Tarabichi et al., 2023). Importantly, anaerobic bacteria, including *Peptostreptococcus* spp., *Finegoldia magna*, and *Bacteroides fragilis*, were seen in higher numbers in the oncologic EPR cohort. Despite this and other mounting evidence that anaerobic bacteria account for a greater proportion of PJI than previously thought, current ICM guidelines do not recommend antibiotic coverage for anaerobic microorganisms (Patel, 2023). Additionally, our study showed a small proportion of Gram-negative bacteria; a study by Sanders et al. (2019) implicated predominantly Gram-negative bacteria in pelvic EPR, highlighting the inconsistency in the orthopedic oncology literature (Sanders et al., 2019).

Even within patients with oncologic EPR, notable differences in microorganism profiles across different sub-cohorts were noted. In particular, no patients with EPR for metastatic bone disease isolated Gram-negative rods, whereas the overwhelming majority isolated CoNS (including *S. epidermidis*), *S. aureus*, or *Enterococcus* spp. Moreover, patients with primary soft tissue sarcomas demonstrated a higher proportion of PJI caused by Gram-negative rods, while primary tumors of bone demonstrated a higher proportion of PJI caused by *Streptococcus* spp. It has been shown that tumors have an intrinsic microbiologic makeup and that the intratumoral composition of microbes may facilitate cancer progression and metastasis through induction of chronic inflammation, activation of carcinogenic pathways, and protection of circulating cancer cells (Yang et al., 2023). In support of this theory, analyses of breast and colorectal cancer have demonstrated a positive relationship between gut dysbiosis and the likelihood of cancer metastasis (Yuan et al., 2022; Zhang et al., 2022). Given that these two populations are likely to have vastly different microbiome profiles, amidst the mounting evidence of the role of microbiome disruption in PJI development, it follows that the respective microbiological profile of PJIs following oncologic EPR would also be different.

Our study also examined the TTP of microorganisms isolated in oncologic PJIs. We found a wide range of TTPs, with *Klebsiella pneumoniae*, *Escherichia coli*, *Enterococcus* spp., *Streptococcus* spp., and *Candida* spp. demonstrating the fastest TTP. This was in contrast with the results from Tarabichi et al. (2023) in their study on traditional total joint arthroplasty, which noted the fastest TTP for methicillin-resistant *Staphylococcus aureus* (Tarabichi et al., 2023). In addition, TTP for *Candida* spp. was notably faster in our study when compared to Tarabichi et al. (2023) (2 d vs. 5.3 d, respectively) and similar for *S. epidermidis* (3 d vs. 4.2 d). The present study also differed from that of Tarabichi et al. (2023) in that the TTP for Gram-positive vs. Gram-negative bacteria was equivalent. Our results demonstrated the slowest TTP in synovial fluid samples, whereas Tarabichi et al. (2023) reported the fastest TTP in synovial fluid. Our study also compares TTP based on culture medium, which was not performed in previous studies and provides additional insight into potential advantages of incubation on both solid media and broth. These variable findings suggest a potential difference in the colonization patterns of microorganisms in oncologic EPR and conventional arthroplasty PJI and highlight the need for larger, multi-institutional analyses on this topic. Importantly, the high proportion of *S. aureus* in the oncologic cohort may shorten overall TTP and bias direct comparisons between oncologic and conventional cohorts.

This study is not without limitations. First, this study is retrospective in nature, carrying inherent biases that cannot be avoided and may confound the analysis. Second, while we presented a diverse profile of microorganisms, our sample size was still relatively small and confined to a single institution. The limited sample size restricts the generalizability of our findings and precludes important subgroup analyses, such as stratification by joint type, infection chronicity, or inclusion of revision arthroplasty patients. Larger, multi-institutional and multi-regional studies are needed to address these limitations and enable more robust, matched analyses. This study also analyzed cases of oncologic EPR PJI over a 22-year period; during that time, there have been several changes in surgical care that may have had an impact on infection characteristics. Similarly, limitations in the available medical record data prevented the collection of potential mediating variables, particularly prior antibiotic use in the oncologic cohort. Additionally, the previous literature has shown an association between RT and the microbiome; thus, it represents a potential confounding bias that more than half of the patients included in this study received prior RT (Liu et al., 2021). Lastly, real-time sample culture data were only available from January 2010 onwards, further limiting our sample for assessing TTP. Nonetheless, this study represents the largest cohort of oncologic PJI with microbiological profiling in the current literature.

Our study found significant variability in the microbiological profile between oncologic EPR and conventional joint arthroplasty, warranting further investigation to confirm these findings and evaluate potential implications for clinical management. The benefits of more targeted antibiotic therapy are 2-fold: decreased systemic side effects from broad-spectrum, non-specific therapy and an increased chance of treatment success. Both are paramount in oncologic populations that, due to ongoing malignant processes and subsequent comorbidities, have a narrower range of tolerability for systemic toxicity and treatment failure. However, given the present study's limitations, the results must be interpreted cautiously; future studies should include larger, multi-institutional cohorts that expand on the influence of mediating variables, such as prior antibiotic use and primary malignancy location, and improve the feasibility and robustness of additional subpopulation analyses. A greater understanding of the microbiologic profile creates an opportunity for improved antibiotic stewardship in an oncologic population that is already at risk for severe complications from infection.

## Data Availability

Raw research data used for this study might be available pending the establishment of data user agreements between institutions.

## Code availability

Codes for the statistical analysis are available upon reasonable request to the study authors.
